# Operation for Strabismus

**Published:** 1841-03

**Authors:** 


### OPERATION FOR STRABISMUS.

This operation was performed at Little Rock, in Arkansas, in January last, as will be seen by the following extract from a newspaper of that place:

"*Novel Surgical Operation.*—Dr. W. J. Goulding of this city, a few days since applied the novel operation of tenotomy to the cure of squinting. The subject was Mr. Isaac M. McCurdy, aged 24 years. His left eye, from early infancy, had been drawn upwards and inwards towards the inner canthus, by the contraction of the internal straight muscle of the eyeball. The eye was relieved from this distortion by retracting the eye-lids and making a small incision of the conjunctiva or outer membrane, near the inner angle of the eye, so as to expose the insertion of the contracted muscle, under which a small probe was introduced and the fibres divided with a pair of small scissors. The eye instantly resumed its normal position, and now acts with the other in all its motions."

